# The Effect of Bifid Triple Viable on Immune Function of Patients with Ulcerative Colitis

**DOI:** 10.1155/2012/404752

**Published:** 2012-08-15

**Authors:** Guohua Li, Sheng Zeng, Wangdi Liao, Nonghua Lv

**Affiliations:** Department of Gastroenterology, The First Affiliated Hospital of Nanchang University, Nanchang 330006, China

## Abstract

*Objective*. To study effect and its mechanism of Bifid Triple Viable for initially treating ulcerative colitis with 5-aminosalicylic acid. *Methods*. 82 patients, who were firstly diagnosed as ulcerative colitis, were randomized into experiment group (41 cases, treated with Bifid Triple Viable and Etiasa) and control group (41 cases, treated with Etiasa). The clinic symptom score, colon mucosa inflammation score, and some immune indices were detected and compared between two groups before and two months after treatment. *Results*. Two months after treatment, the clinical symptom score, colon mucosa inflammation score, and IL-1**β** expression in colon mucosa decreased significantly (*P* < 0.01), and IL-10 and IgA expressions in colon mucosa increased significantly (*P* < 0.01). Those differences were more marked in experiment group than control group (*P* < 0.05). However, peripheral blood T cell subgroup, immunoglobulins, and complements had no significant difference between two groups two months after treatment, but the ratio of peripheral blood CD4+ T cell to CD8+ T cell in experiment group increased more than that in control group (*P* < 0.05). *Conclusion*. Bifid Triple Viable contributed to Etiasa to treat ulcerative colitis in inducing remission period, which was perhaps related to affecting the patient's immune function.

## 1. Introduction

The ulcerative colitis (UC) is a serious health care problem of unknown etiology, which affects 0.1% of the population in Asia-Pacific area [1]. The morbidity of UC in Asia-Pacific area is lower than western countries, but has an increasing trend in the last few years. UC is a chronic immune-mediated disease. Many studies confirmed that endogenous bacteria played an important role [2–5]. Inflammatory colitis couldn't be induced or developed in experimental animals when the intestinal lumen of animals did not contain the bacterial flora. On the other hand, the bacterial species in the intestinal lumen might promote or inhibit intestinal inflammation [6–8]. Many studies showed the preparations containing bifidobacteria, lactobacilli, or Escherichia coli could reduce intestinal inflammation. A new therapy of UC based on probiotic preparations that usually contain those strains is going to use [9–11]. Bifid Triple Viable, the combination of bacillus acidophilus, bifidobacterium bifidum and fecal streptococci, made in Shanghai Xinyi pharmacy Inc. of China, was usually used in patients with diarrhea, alteration of intestinal flora [12, 13]. It has not been known if it is safe and valid in treating UC with 5-ASA.

The beneficial effect of probiotics is demonstrated mainly in pouchitis and UC [14–17]; however, their mechanisms are not well confirmed. Several recent human studies involved the effect of probiotic intake on immune function. Those suggested that some probiotic strains could modulate both natural and acquired immune responses. If healthy human adults were administered fermented milk including specific strains of probiotics (*Bifidobacteriumlactis* Bb12 or* Lactobacillus johnsonii* La1) for three weeks, the phagocytic capacity of their peripheral blood leucocytes (monocytes and polymorphonuclear) increased [18]. After cessation of this probiotic consumption, the granulocytes enhanced higher in phagocytic cell function compared with monocytes [19–22]. The expressions of receptors such as CR1, CR3, Fc*γ*RI and FcR in neutrophils increased significantly [23]. Schultz et al. [24] observed that *Lactobacillus GG* fermented milk enhanced both rotavirus specific and non-specific antibody responses. The infants fed with *LactobacillusGG* had higher IgG, IgA, and IgM immunoglobulin secreting cells compared with those given a placebo.

Although probiotic intake enhanced human immune function in healthy persons, the effect of probiotic intake on immune function of patients with UC had not yet been well reported. Matsumoto and colleagues [25] found that Lactobacillus casei strain Shirota improved murine chronic inflammatory bowel disease, which was associated with the down-regulation of interferon gamma (IFN-gamma) and IL-6 production in mononuclear cells of large intestinal lamina propria. Therefore the probiotics intake may have the similar effect on immune function of patients with UC. So we hypothesized that probiotic intake affect immune function of patients with UC. In this paper, we evaluated the effects of probiotic preparation Bifid Triple Viable (Shanghai Xinyi pharmacy Inc., China) on immune function through measuring the expression of peripheral blood immunoglobulins, complements, and T cell subgroups, and colon mucosa IL-1*β*, IL-10 and IgA protein expressions in patients with UC.

## 2. Materials and Methods

### 2.1. Patients and Samples

Patients, who were diagnosed and treated firstly in department of gastroenterology, the first affiliated hospital of Nanchang university from November of 2004 to June of 2006 according to UC diagnosis cretirion1, were randomized successively into experiment group and control group according to random digits table. The patients in experiment group, including 41 cases (17 women and 24 men; mean age 43 years; range from 22–70 years), were treated with Bifid Triple Viable (2 capsules three times daily) and 5-aminosalicylic acid (1 g two times daily) (5-ASA, Etiasa, Ethypharm Industries, France). Those in control group, including 41 cases too (18 women and 23 men; mean age 42.5 years; range from 21–67 years), were treated with 5-ASA. The patients, who received drugs treatment in two weeks or had other organic diseases, were excluded. The colon mucosa samples in the same colon segment, and peripheral blood were collected from each patient before and two months after treatment. The colon mucosa samples were fixed in 4% paraformaldehyde, and embedded in paraffin for immunohistochemistry and histological assay. Each patient had been monitored blood routine, urine routine, stool routine, erythrocyte sedimentation rate (ESR), hepatic function, renal function, stool bacteria culture, temperature, and symptoms each two week after treatment. They were monitored until they were normal.

### 2.2. Clinical Symptom and Histology Assessment

The clinical symptom and colon mucosa inflammation degree of the patients with UC were assessed respectively. UC were classified into mild, moderate, and severe UC according to Montreal classification [26, 27]. The total score of clinical symptoms was calculated in each group according to Mayo Score for UC [28].

One 5 *μ*m thick section, collected from each of those colon specimens, was stained by hematoxylin and eosin for scoring inflammation degree ranging from 0 to 4 according to Ameho's method [29]. The grade from 0 to 4 was scored 0, 1, 2, 3, and 4 respectively. The total score of colon mucosa inflammation degree was calculated in each group.

### 2.3. Detecting Peripheral Blood Immunoglobulins, Complements and T-Cell Subgroup

Peripheral blood T-cell subgroup of each patient with UC was detected by flow cytometrey (FACSCalibour, Becton Dickinson, USA). The peripheral blood immunoglobulins, complement C3 and C4 were detected by Immunoglobulin- Analysator (Array 360 System, Beckman, German).

### 2.4. Immunohistochemistry

Each sample of colon mucosa embedded in paraffin was sectioned three 5 *μ*m thick section to detect IL-1*β*, IL-10 and IgA expressions by immunohistochemistry respectively. Immunohistochemistry test was performed according to the procedure of SP9000 kit (Beijing Zhongshan Co. Beijing, China). The IgA primary antibody (goat IgA polyclonal antibody, Beijing Zhongshan Co. Beijing, China) was diluted 1 : 500 by 0.01 mol/L phosphate buffer saline (PBS). The IL-1*β* primary antibody (goat IL-1*β*monoclonal antibody, R&D Systems Inc. Minneapolis, USA) was diluted 1 : 200 by 0.01 mol/L PBS. The IL-10 primary antibody (mouse IL-10 monoclonal antibody, R&D Systems Inc., Minneapolis, USA) was diluted 1 : 200 by 0.01 mol/L PBS. The test set negative controls by replacing the primary antibody with normal rabbit serum under the same experimental conditions. The positive particle showed dark brown under microscopy. The average number of positive particles in each field was calculated by averaging the number of positive particles of five random fields in each slide under microscopy in 400 magnifications. The total positive particles for IgA, IL-1*β*, or IL-10 antibody in each group were calculated respectively.

### 2.5. Statistical Analysis

SPSS 10.0 for windows (Chicago, IL, USA) was used to do the statistical analysis. Measurement data were reported as mean ± SD, and *t*-test was used to investigate the differences between two groups. Chi-square analysis or Wilcoxon rank-sum test was also used for the assessment of Enumeration data.

## 3. Results

### 3.1. Clinical Features of Patients with UC

Patients, who were enrolled into our study, were diagnosed firstly in our hospital, and all were in active stage. The clinical features of these patients listed in Table 1. There was no significant difference between experiment group and control group in defecation frequency, hemafecia quantity, course of disease, extent of lesion under colonoscopy, and clinical grade of UC.

### 3.2. Clinical Symptoms and Colon Mucosa Inflammation Assessment

There were no significant differences in the total scores of clinical symptoms and colon mucosa inflammation between two groups before treatment. Two months after treatment, the total scores of colon mucosa inflammation and clinical symptoms decreased significantly in each group (*P* < 0.01, Table 2). Moreover, those scores of colon mucosa inflammation and clinical symptoms in experiment group were lower than those in control group (*P* < 0.01).

Five patients, 2 from experiment group and 3 from control group, had white blood cells descending two months after treatment, but they were above 3.0 × 10^9^/L. The white blood cells in those patients recovered two week after stopping using 5-ASA.

### 3.3. Peripheral Blood Immunoglobulins, Complements and T-Cell Subgroup

**Figure 1:**
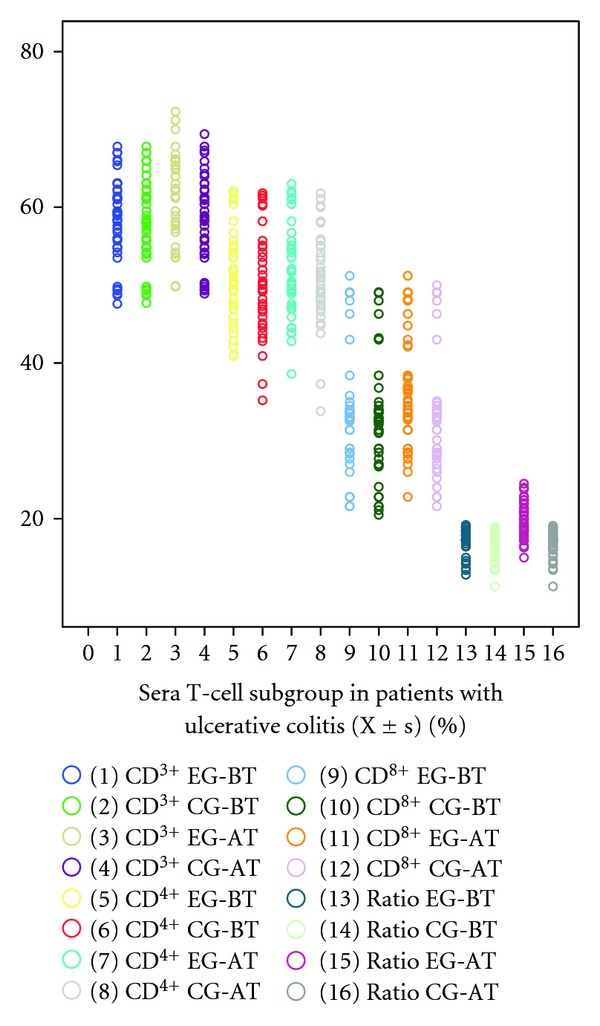
EG stands for experiment group; CG stands for control group; BT stands for “before treatment”. AT stands for “after treatment”. Ratio stands for the tenfold ratio of CD4^+^T cells to CD8^+^ T cells. There was no significant difference regarding the average values of T-cell subgroup between two groups before treatment and two months after treatment (*P* > 0.05). However, the ratio of CD4^+^ T cell to CD8^+^ T cell in experiment group two months after treatment increased more than that in control group (*P* < 0.05).

There were no significant differences regarding the average values of peripheral blood immunoglobulins, complement C3 and C4, and T-cell subgroup between two groups before treatment (*P* > 0.05, Table 3, Figure 1). Two months after treatment, there was no significant alternation in the average values of peripheral blood immunoglobulins, complement C3 and C4, and T-cell subgroup (*P* > 0.05). However, the ratio of CD4^+^ T cell to CD8^+^ T cell in experiment group increased more than that in control group (*P* < 0.05).

### 3.4. Expressions of IgA, IL-1*β*, and IL-10 in Colon Mucosa

**Figure 2:**
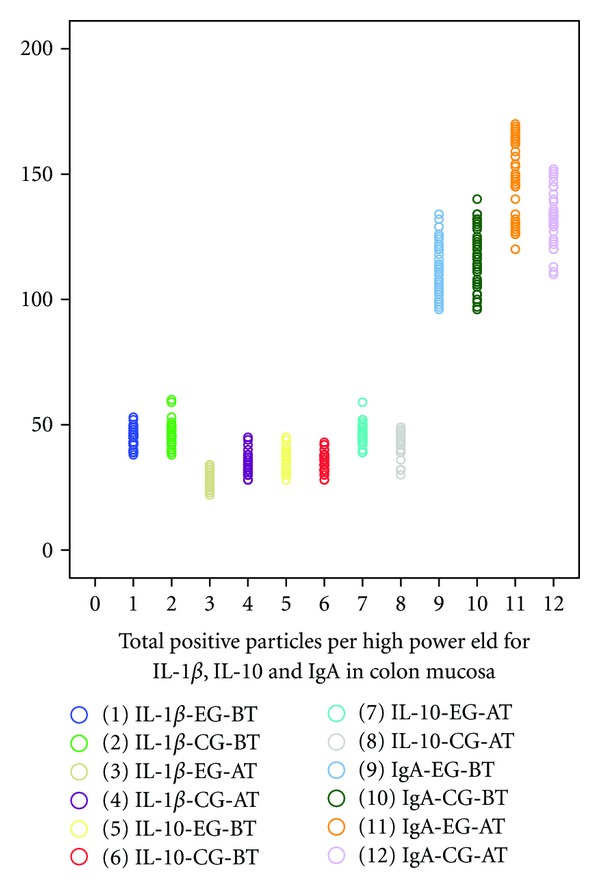
EG stands for experiment group; CG stands for control group; BT stands for “before treatment”. AT stands for “after treatment”. The average number of positive immunoreactivity particles for IgA, IL-1*β*, or IL-10 in colon mucosa was no significant difference before treatment between two groups. Two months after treatment, the average number of positive particles for IgA and IL-10 in colon mucosa in each group increased significantly (*P* < 0.01). Moreover, the average number of positive particles for IgA and IL-10 in experiment group was more than that in control group (*P* < 0.01). However, average number of positive particles of IL-1*β* was opposite to that of IgA and IL-10 (*P* < 0.05).

**Figure 3:**
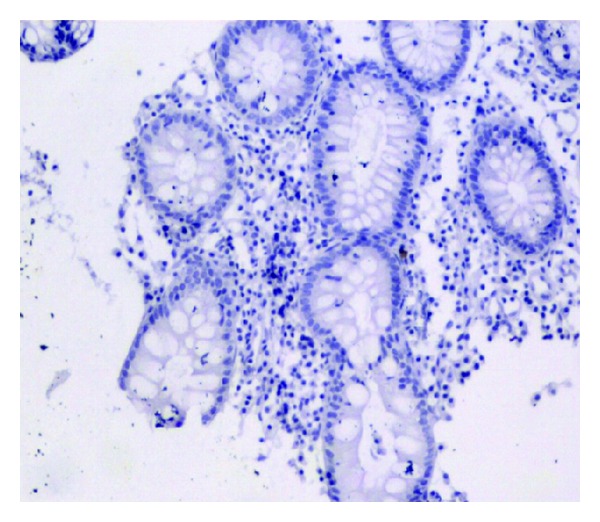
No positive particle (1 × 100).

**Figure 4:**
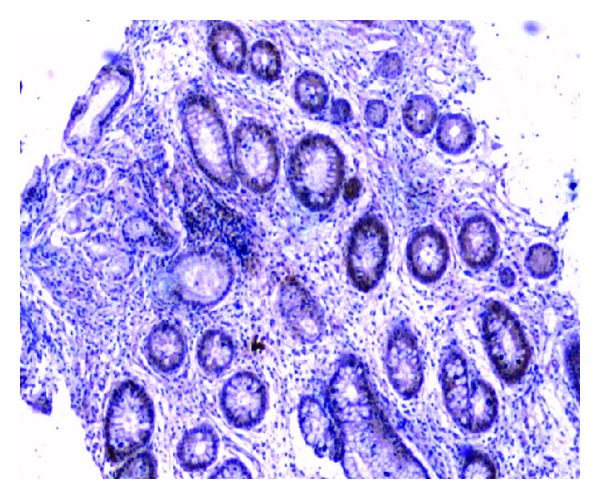
Positive particle for anti-IL-10 (1 × 40).

**Figure 5:**
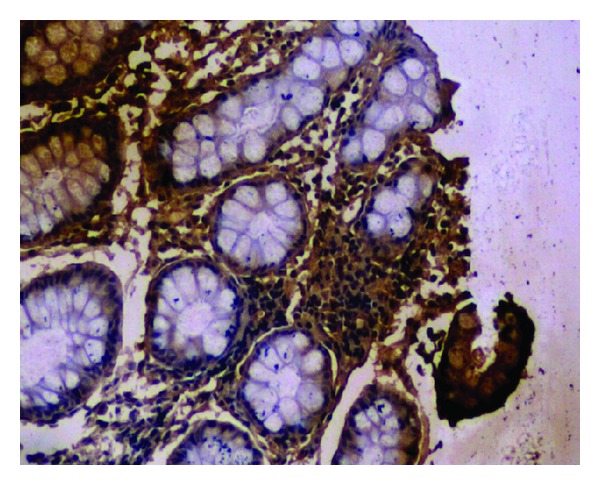
Positive particle for anti-IgA (1 × 100).

**Figure 6:**
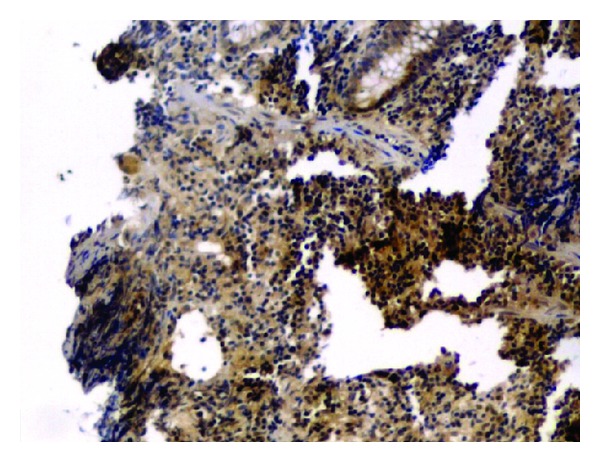
Positive particle for anti-IL-1*β* (1 × 40).

The average number of positive immunoreactivity particles for IgA, IL-1*β*, or IL-10 in colon mucosa was no significant difference before treatment between two groups. Two months after treatment, the average number of positive particles for IgA and IL-10 in colon mucosa in each group increased significantly (*P* < 0.01, Figure 2). Moreover, the average number of positive particles for IgA and IL-10 in experiment group was more than that in control group (*P* < 0.01). However, average number of positive particles of IL-1*β* was opposite to that of IgA and IL-10 (*P* < 0.05, Figures 3, 4, 5 and 6).

## 4. Discussion

Intestinal microflora played an important role in the pathogenesis of UC. Many studies 30–33 supported that probiotics could contribute to cure of inflammatory bowel disease. The probiotic preparations could prevent pouchitis onset, and chronic pouchitis relapse. But the risk of bacterial translocation should be evaluated carefully [30–33]. A healthy host has the integrity of the intestinal barrier, and has a low rate of bacterial translocation. When the intestinal barrier is not integrated, or when the immune function is weakened by some diseases, the pathogenic bacteria in intestinal lumen can transit through intestinal wall, and therefore cause septicemia [34]. So, it is necessary to confirm probiotic safety in inflammatory bowel disease (IBD) especially. Bifid Triple Viable, the combination of bacillus acidophilus, bifidobacterium bifidum and fecal streptococci, made in Shanghai Xinyi Inc.of China, were usually used in patients with diarrhea, alteration of intestinal flora [12, 13]. We have not yet known if it is safe and feasible for treating UC. In this paper, we found that the clinical symptoms and colon mucosa inflammation of patients with UC alleviated more markedly in experiment group than that in control group. That was to say, the treatment effectiveness with Bifid Triple Viable and Etiasa for UC was better than with Etiasa only. Although some patients had WBC descending, there was no significant difference between two groups. Moreover WBC recovered in 2 weeks after stopping using Etiasa. We thought the WBC descending was related to Etiasa, but not Bifid Triple Viable. On the other hand, we did not find septicemia in two groups during treatment period for two months. It explained that Bifid Triple Viable is safe and feasible for treating UC with Etiasa. However, most enrolled patients (86.6%) in our study belonged to mild or moderate grade UC patients, and all patients were treated initially. The safety and availability for treating severe UC or maintaining treatment may require further research. Our study was not double-blind. In order to reduce the experiment bias as far as possible, we assigned different researchers to do clinical treatment, immunohistochemistry, and statistics respectively. It has been known that the UC treatment requires several years, and inducing remission period may be several months [35–38]. So we selected two months as experiment period in order to observe the safety and validity in inducing remission treatment of initial UC patients.

The beneficial effect of probiotics was demonstrated mainly in pouchitis and ulcerative colitis. However, their mechanisms of action were not well understood. In recent healthy human studies, there was strong evidence to suggest that some probiotic strains could modulate both natural and acquired immune responses of healthy human 44–46_. _Although probiotic intake enhanced healthy human immune function, there was little understanding about the effect of probiotic intake on immune function of patients with ulcerative colitis. Matsumoto and colleagues [25] found that Lactobacillus casei strain Shirota (LcS) improved murine chronic inflammatory bowel disease, which was associated with the down-regulation of IFN- gamma and IL-6 production in mononuclear cells of large intestinal lamina propria. Therefore the probiotics intake may have similar effect on immune function of patients with ulcerative colitis. In this paper, we detected peripheral blood immunoglobulins, complement C3 and C4, and T cell subgroups, and colon mucosa IL-1*β*, IL-10 and IgA expressions in patients with UC. We found that the IL-10 and IgA expressions in colon mucosa, the ratio of peripheral blood CD4^+^ T cell to CD8^+^ T cell in experiment group increased more than those in control group, but the IL-1*β* was opposite. Therefore Bifid Triple Viable affected the immune functions of patients with UC. However, we did not found that Bifid Triple Viable affected the peripheral blood immunoglobulins, complement C3 and C4. The possible reasons may be: (1) the quantity of Bifid Triple Viable was small; (2) the treatment time of using Bifid Triple Viable was short; (3) Bifid Triple Viable may affect mainly mucosa immune functions. Those need assess further by many randomized, double-blind, controlled studies. The reasons we detected mucosa IgA, IL-1, and IL-10 expressions, and peripheral blood immunoglobulins, complements, and the ratio of CD4^+^ T cells to CD8^+^ T cells in this study were: (1) mucosa IgA is an important immunoglobulin, which reduce mucosa bacterial translocation [39, 40]. (2) IL-1 is a pro-inflammatory cytokine [41], but IL-10 is an anti-flammatory cytokine [42]. They may reflect mucosa inflammatory condition and trend. (3) immunoglobulins reflect acquired immune fuctions, and the ratio of CD4^+^ T cells to CD8^+^ T cells reflects T1 type or T2 type immune response tread [43].

## 5. Conclusion

This study is first to report the effect of Bifid Triple Viable on immune function in patients with ulcerative colitis. We found that Bifid Triple Viable contributed to Etiasa to treat ulcerative colitis in inducing remission period, which was perhaps related to affecting the patient's immune function.

## Figures and Tables

**Table 1:** Clinical features of patients with UC.

Group	*n*	Defecation frequency	Hemafecia (mL/d)	Disease course (month)	Extend (*n*)			Grade (*n*)		
					Rectum	Left colon	Extensive colon	Mild	Moderate	Severe
Experiment group	41	4 ± 1.8	22 ± 3.8	14 ± 2.4	14	22	5	13	22	6
Control group	41	4 ± 2.0	21 ± 3.6	15 ± 2.5	15	22	4	15	21	5
Statistic value	—	*z* = 0.256	*t* = 1.223	*t* = 1.885	*χ* ^2^ = 0.146			*χ* ^2^ = 0.257		
*P* value	—	0.877	0.229	0.085	0.930			0.879		

*z* value calculated by Wilcoxon rank-sum test, *t* value calculated by *t*-test, and *χ* ^2^ value calculated by Chi-square analysis between two group.

**Table 2:** Clinical symptom score and colon mucosa inflammation score ($\overset{-}{x}$ ± *s*).

Group	*n*	The score of clinical symptoms (Mayo score)		The score of colon mucosa inflammation	
		Before treatment	After treatment	Before treatment	After treatment
Experiment group	41	7.17 ± 1.26	2.46 ± 0.67^∗^	2.07 ± 0.29	0.54 ± 0.14^∗^
Control group	41	7.02 ± 1.21	3.96 ± 0.71^∗^	2.02 ± 0.29	0.71 ± 0.17^∗^
*z* value		0.560	2.654	0.784	2.742
*P* value		0.674	0.008	0.431	**0.010**

*z* value calculated by Wilcoxon rank-sum test between two groups. *P < 0.01, comparing with before treatment using Wilcoxon rank-sum test.

**Table 3:** Peripheral blood immunoglobins and complements of patients with UC ($\overset{-}{x}$ ± *s*, g/L).

Group	*n*	IgG		IgA		IgM		C3		C4	
		BT	AT	BT	AT	BT	AT	BT	AT	BT	AT
Experiment group	41	12.7 ± 2.8	12.3 ± 2.4	2.36 ± 0.84	2.26 ± 0.76	1.56 ± 0.73	1.43 ± 0.70	1.08 ± 0.34	1.03 ± 0.33	0.31 ± 0.12	0.30 ± 0.11
Control group	41	13.2 ± 2.9	12.9 ± 2.8	2.48 ± 1.23	2.42 ± 1.01	1.51 ± 0.80	1.48 ± 0.72	1.06 ± 0.28	1.02 ± 0.27	0.27 ± 0.10	0.27 ± 0.11
*t* value	—	0.794	1.042	0.516	0.811	0.296	0.319	0.291	0.311	1.640	1.235
*P* value	—	0.430	0.299	0.633	0.419	0.765	0.756	0.77	0.742	0.106	0.226

BT stands for “before treatment”. AT stands for “two months after treatment”. The statistics using *t*-test.
